# Changes in transcriptomic landscape with macronutrients intake switch are independent from O-GlcNAcylation levels in heart throughout postnatal development in rats

**DOI:** 10.1016/j.heliyon.2024.e30526

**Published:** 2024-04-30

**Authors:** Antoine Persello, Thomas Dupas, Amandine Vergnaud, Angélique Blangy-Letheule, Virginie Aillerie, Angélique Erraud, Yannick Guilloux, Manon Denis, Benjamin Lauzier

**Affiliations:** a Nantes Université, CNRS, INSERM, l'institut du thorax, F-44000, Nantes, France; b Nantes Université, Inserm UMR 1307, CNRS UMR 6075, Université d’Angers, CRCI2NA, F-44000, Nantes, France; c Nantes Université, CHU Nantes, CNRS, INSERM, l'institut du thorax, F-44000, Nantes, France

**Keywords:** O-GlcNAcylation, Post-translational modification, Transcriptomic, Development, Macronutrients switch

## Abstract

**Background:**

Dietary intake and metabolism variations are associated with molecular changes and more particularly in the transcriptome. O-GlcNAcylation is a post-translational modification added and removed respectively by OGT and OGA. The UDP-GlcNAc, the substrate of OGT, is produced by UAP1 and UAP1L1. O-GlcNAcylation is qualified as a metabolic sensor and is involved in the modulation of gene expression. We wanted to unveil if O-GlcNAcylation is linking metabolic transition to transcriptomic changes and to highlight modifications of O-GlcNAcylation during the postnatal cardiac development.

**Methods:**

Hearts were harvested from rats at birth (D0), before (D12) and after suckling to weaning transition with normal (D28) or delayed weaning diet from D12 to D28 (D28F). O-GlcNAcylation levels and proteins expression were evaluated by Western blot. Cardiac transcriptomes were evaluated via 3′SRP analysis.

**Results:**

Cardiac O-GlcNAcylation levels and nucleocytoplasmic OGT (ncOGT) were decreased at D28 while full length OGA (OGA) was increased. O-GlcNAcylation levels did not changed with delayed weaning diet while ncOGT and OGA were respectively increased and decreased. *Uapl1* was the only O-GlcNAcylation-related gene identified as differentially expressed throughout postnatal development.

**Conclusion:**

Macronutrients switch promotes changes in the transcriptome landscape that are independent from O-GlcNAcylation levels. UAP1 and UAP1L1 are not the main regulator element of O-GlcNAcylation throughout postnatal development.

## Highlights

• High fat diet impacts gene expression without changes O-GlcNAc levels.
• UAP1L1 is the only HBP-related gene differentially expressed during development.
• UAP1 and UAP1L1 are not the main regulator element of O-GlcNAc levels.
• OGT and OGA and UAP1 are not regulated at transcriptional level.
• O-GlcNAcylation is a finely regulated mechanism throughout postnatal development.

## 1 Introduction

**Fig. 1:**
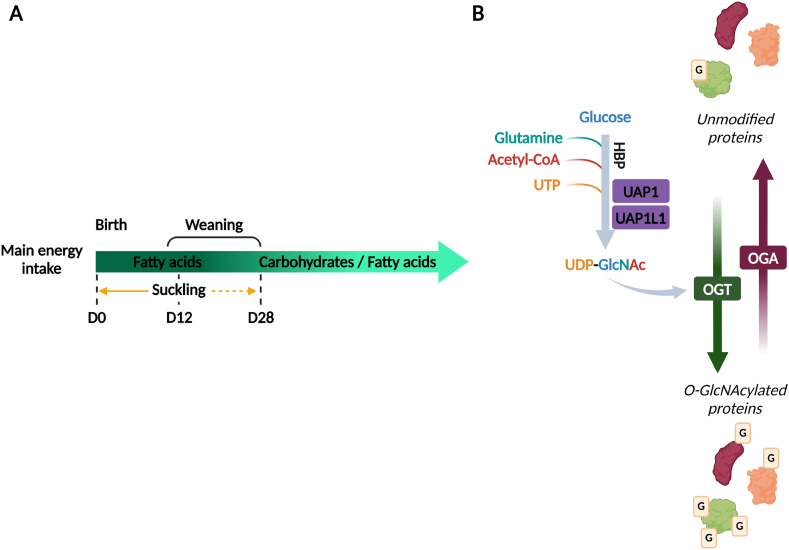
Main sources of energy, O-GlcNAcylation and hexosamine biosynthesis pathway (**A**) Variation of energy sources throughout development. (**B**) O-GlcNAcylation and the hexosamine biosynthesis pathway. Created with BioRender.com.

Organ metabolism depends on availability of metabolites and substrates, metabolic pathways and their regulation, and on the activity of related enzymes. Metabolism evolves with the nutrient intake from the earliest embryonic developmental stage throughout lifespan, more particularly in the heart that shows a high sensitivity to substrate availability. Lipids in the maternal milk are the main energy supply afterbirth [1]. At this stage, the heart has a high energy demand due to structural and functional modifications resulting in the increase in mitochondrial mass to provide sufficient adenosine triphosphate (ATP) *via* the oxidative phosphorylation of the lipids [2,3]. The suckling to weaning transition leads to a change in the nutrient intake and is responsible for a change of metabolic substrate with the maternal milk replaced by a mixed diet. It results in the modification of the share of substrates for ATP production. After birth, 60–90 % of the cardiac ATP is generated from lipids and 10–40 % from carbohydrates (Fig. 1A). Moreover, the suckling to weaning transition is associated with molecular changes of glucose metabolism [4,5], that are partly ensured by changes in the transcriptomic landscape [6,7]. Expression of genes involved in ribosome and cell cycle pathways decreases throughout postnatal development, while genes associated with metabolism (e.g. mitochondria and oxidation-related pathways) and cardiac specific pathways are more expressed with the maturation of the heart [8]. Substrate availability also regulates transcription factor and can impact transcription processes [9]. Although metabolism and gene expression are significantly modified during postnatal cardiac development, the link between metabolic transition and transcription modulation remains poorly understood.

O-GlcNAcylation (O-GlcNAc) is a post-translational modification dependent of cell metabolism notably involved in transcription and metabolic reprogramming [[10], [11], [12]] [[10], [11], [12]] [[10], [11], [12]]. The addition and the deletion of the GlcNAc moiety is a dynamic and reversible process involving the O-GlcNAc transferase (OGT) and the O-GlcNAcase (OGA) respectively [13]. OGT and OGA are respectively encoded by one gene, but several isoforms exist due to alternative splicing. OGT exists in 3 isoforms: nucleocytoplasmic, mitochondrial, and short OGT (ncOGT, mOGT and sOGT respectively), while two isoforms were identified for OGA: full length and short OGA (OGA and sOGA respectively) [14]. The uridine diphosphate N-acetylglucosamine (UDP-GlcNAc) is the end-product of the hexosamine biosynthesis pathway (HBP) and the sugar donor of OGT. Due to the participation of several metabolites (glucose; acetyl-CoA; glutamine and uridine triphosphate) in the synthesis of the UDP-GlcNAc, the HBP is defined as a metabolic hub [12] (Fig. 1B). Even if O-GlcNAcylation is defined as a nutrient driven process, its regulation is not completely understood and remains a major challenge [15].

The aims of this study are to (1) unveil the potential role of O-GlcNAcylation as a link between the transcriptome changes due to metabolic switch following diet transition afterbirth and (2) highlight transcriptome changes along the development and focus on its potential link with the regulation of O-GlcNAcylation.

## 2 METHODS

### 2.1 Animal model

Animal care unit held rats in regulated and controlled temperature (21–24 °C), humidity (40–60 %) and 12 h light/dark cycle, food and water were available *ad libitum*. Protocols were performed in respect with the guidelines and recommendations from the local ethics committee in charge of animal experimentation (Pays de la Loire, France), the French law on animal welfare, the EU Directive 2010/63/EU for animal experiments, and the National Institutes of Health (NIH) Guide for the Care and Use of Laboratory Animals (NIH Pub. No. 85-23, revised 2011).

**Fig. 2:**
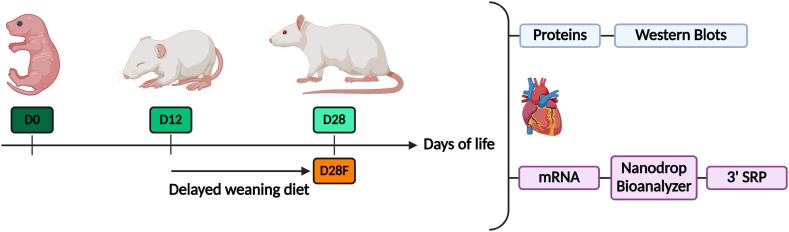
Animal groups and analyses performed. Wistar rats were sacrificed after birth (D0), before (D12) and after the weaning (D28) and after the weaning with delayed weaning diet (D28F). Created with BioRender.com.

Pregnant Wistar rats were received at the Unité Thérapeutique Expérimentale at 15 days of gestation. Newborn rats were randomly assigned to one of the groups, independently of their weight and size, sex, the latter was determined either by PCR targeting the *Sry* and *Actb* gene (Table 1) (D0 for western blot and qPCR analyses) or by the expression of *Ddx3x* or *Ddx3y* via transcriptomic data (D0 for transcriptomic, D12, D28 and D28F) [16,17]. Rats were used at different developmental ages: right after birth (D0), before (D12; n = 8; 6 males and 2 females) and after weaning (D28; n = 8; 7 males and 1 females). At D12, 8 rats were fed with standard diet (SAFE A04; Safe, France) (D28) and 8 rats were fed with a delayed weaning diet (50 % fat, 30 % protein, 3 % carbohydrate, minerals, and cellulose for the remaining percentage) (#U8954P, 00001 version; Safe, France) (D28F; n = 9; 6 males and 3 females). This diet mimicking mother's milk composition was used to evaluate O-GlcNAc and transcriptomic changes without metabolic modifications associated with the suckling to weaning transition [18] (Fig. 2). Due to the small amount of biological material, animals on D0 were randomly assigned either i) transcriptomic analysis (n = 6; 5 males, 1 female) or ii) western blot and qPCR analyses (n = 8; 7 males and 1 female).

**Table 1:** Primers used for sex determination by PCR.

Genes studied	Forward primer sequence	Reverse primer sequence
*Sry*	TACAGCCTGAGGACATATTA	GCACTTTAACCCTTCGATGA
*Actb*	AGCCATGTACGTAGCCATCC	TGTGGTGGTGAAGCTGTAGC

### 2.2 Heart harvesting

Ansthesia of rats was achieved by inhalation of an isoflurane/air mixture (Forène, Abbott, Rungis, France; induction phase: 5 % isoflurane, with an air flow rate of 1 L/min; maintenance: 2 % isoflurane with an air flow rate of 0.5 L/min). Under anesthesia , hearts were harvested and flash-frozen in liquid nitrogen to preserve post-translational modifications for subsequent biochemical and molecular biology analyses. The biocollection procedure adhered to standardized conditions, with samples collected between 8:30 a.m. and 10:00 a.m., mitigating potential circadian influences on protein O-GlcNAcylation levels.

### 2.3 Total protein and RNA extraction from hearts

Frozen hearts were crushed to obtain powder for protein and RNA analyses as previously described [19]. All steps were carried out in liquid nitrogen to preserve post-translational modifications and RNA integrity. Protein and RNA extraction from heart powder was processed as previously described [19]. RNA was recovered in sterile RNAse free water (Qiagen, Germany), dosed with the NanoDrop ND-1000 Spectrophotometer (ThermoFisher Scientific, USA) and quality was controlled with RIN measures (Bioanalyzer, Agilent Technologies, USA). Powder, proteins, and RNA extractions were conserved at −80 °C.

### 2.4 Western blot

The O-GlcNAc western blot protocol was validated in previous studies [[19], [20], [21], [22]] [[19], [20], [21], [22]] [[19], [20], [21], [22]]. For consistency purposes, relevant points must be taken into consideration before western blotting. 1) Hearts were collected as quickly as possible and instantly preserved in liquid nitrogen to maintain O-GlcNAcylation; 2) Tissue reduction into powder was performed manually in liquid nitrogen to preserve O-GlcNAcylation; 3) The lysis buffer used was T-PER-based (complete composition is described in Ref. [19]). All samples included were lysed in the same fashion. Protein extraction was performed on ice at all step and centrifuge temperature was set to 4 °C; 4) Samples were kept at −80 °C, from the organ collected to extracted proteins. Additionally, protein from the samples presented were extracted concurrently in lysis buffer and diluted at 2.5 μg/μL.

Stain free gels (#4568086, Bio-Rad) were loaded with a mixture of 25 μg of protein (10 μL at 2.5 μg/μL) and 2.5 μL of Laemmli 5X. Migration was performed in two phases, 15 min at 80 mV followed by 1 h and 30 min at 120 mV (Until the 30 kDa molecular weight marker reach the bottom of the gel (#G623, Opti-Protein Ultra Marker, Applied Biological Materials). After migration, proteins have been transferred on a nitrocellulose membrane using Trans-Blot® Turbo™ Transfer System technology (High molecular weight preset). The stain-free image was taken immediately after transferred using the ChemiDoc XRS + System (Bio-Rad). Membrane was saturated under soft agitation at room temperature with 3 % BSA or 5 % milk, depending on the antibody used (see Table 2). For O-GlcNAc, the membrane was then incubated 1 h at room temperature with the primary antibody diluted in 3 % BSA, washed (3*10 min) with TBS-0.1 % tween 20 and revealed using the Clarity Western ECL substrate (#1705061, Bio-Rad) and the ChemiDoc XRS + System. For OGT, OGA, UAP1, UAP1L1, the membrane was incubated overnight at 4 °C with the primary antibody diluted in 5 % milk, washed (3*10 min) with TBS-0.1 % tween, incubated 1 h at room temperature with the secondary antibody diluted in 5 % milk, washed again with TBS-0.1%tween 20 and revealed using the Clarity Western ECL substrate and the ChemiDoc XRS + System. Finally, densitometric analyses were performed using Bio-Rad's Image Lab software (Image Lab 6.1), and background reduction was applied (Supplemental Data).

**Table 2:** Antibodies used in western blot experiments.

Target	Primary antibody		Secondary antibody	
	Antibody	Dilution	Antibody	Dilution
O-GlcNAc	HRP Anti-O-linked N-Acetylglucosamine [RL2] Abcam #ab201995	1/10 000^#^	/	/
OGT	OGT (D1D8Q) Cell Signaling #24083	1/400*	Anti-rabbit IgG, HRP-linked antibody Cell Signaling #7074	1/10000*
OGA	Anti-MGEA/OGA Abcam #ab105217	1/5000*	Anti-rabbit IgG, HRP-linked antibody Cell Signaling #7074	1/10000*
UAP1	UAP1 Proteintech #16318-1-AP	1/2000*	Anti-rabbit IgG, HRP-linked antibody Cell Signaling #7074	1/10000*
UAP1L1	UAP1L1 Proteintech #25262-1-AP	1/1000*	Anti-rabbit IgG, HRP-linked antibody Cell Signaling #7074	1/10000*

Dilutions carried out in 3 % Bovine Serum Albumin (^#^) or in 5 % milk (*).

### 2.5 Reverse transcription and DGESeq processing and analyses

Complementary DNA (cDNA) was obtained by reverse transcription of isolated RNA using the cDNA Reverse Transcription High-Capacity kit (Applied Biosystem, France). The reverse transcription was carried out in two steps: 10 min at 25 °C followed by 2 h at 37 °C. DGESeq processing and analyses were carried out as described above, following the protocol of Charpentier *et al.* and with the use of the R packages *"clusteRprofiler"* and *"DESeq2"* [21,23,24]. Genes were considered differentially expressed (DEGs) if selection criteria were met Log2 fold change < −0.8 or > 0.8 and Benjamini-Hochberg adjusted p value < 0.05, results are presented in supplemental files (Supplemental Table 1 and Supplemental Table 2). DEGs selected by two-by-two comparisons were consolidated in a list and used to prepare a heatmap (*"pheatmap”* R package) representing the genes expression across all four groups and all samples. Hierarchical clustering based on distance was performed (*“hclust”* R package) to obtain 8 clusters representative of different gene expression profiles. Average and standard deviation of genes expression for each cluster and group, represented as boxplots allowed to visualize the gene expression profile of each cluster across the different groups. Enrichment analysis of GO (2024-03-28 release), KEGG (release 110.0), and Reactome (release 88) allowed us to attribute main involved processes and pathways for each profile.

### 2.6 Quantitative Polymerase Chain Reaction

Polymerase Chain Reaction was performed as described previously with a reaction mixture containing Power SYBR® Green PCR Master mix (#4367659, Applied Biosystem, UK) and primers for the genes of interest (Table 3) [25]. Samples were quantified in duplicate and normalized to housekeeping genes (*Ywhaz* and *Gapdh*). The results were obtained using StepOnePlus software (ThermoFisher Scientific, USA).

**Table 3:** Primers used in qPCR experiments.

Genes studied	Forward primer sequence	Reverse primer sequence
*Uap1*	CAATGCTGGGGGACACTTCA	GACTTTCCATTTGTAGCACGGGG
*Uap1l1*	TCCCGCAGGTGGTTGAATAC	GGGCTCAAACTCCCTGATGA
*Gapdh*	TGATGGCATGGACTGTGG	CAGCAATGCATCCTGCAC
*Ywhaz*	AGCGAGGGACATCTGCAAC	CTTTGCTTTCTGGCTGCGAA

### 2.7 Statistical analyses

Numbers were chosen to reach sufficient statistical power to interpret the results. They were chosen based on the statistical tests used. Results are expressed as an average ± SEM of n different rats. Analyses of Western blots are expressed in relation to the average of the stain free and then normalized to the average of the control samples (D0 for D0-D12-D28 Western blots and D28 for D28-D28F Western blots). A D'Agostino & Pearson test was used for testing normality. Western blots data presenting more than 2 groups were analyzed by a One-way ANOVA with Fisher's LSD test if normality test passed: a Kruskal-Wallis test with uncorrected Dunn's test if not. Western blot used for the comparison of 2 groups were analyzed with unpaired *t*-test if normality passed; otherwise, a Mann-Whitney test was used. A value of p < 0.05 was considered significant. Statistical calculation and graphs were performed with GraphPad Prism software (version 8.4.2) and R (version 4.1.2).

## 3 Results

### 3.1 O-GlcNAcylation levels decrease in the heart throughout postnatal development

**Fig. 3:**
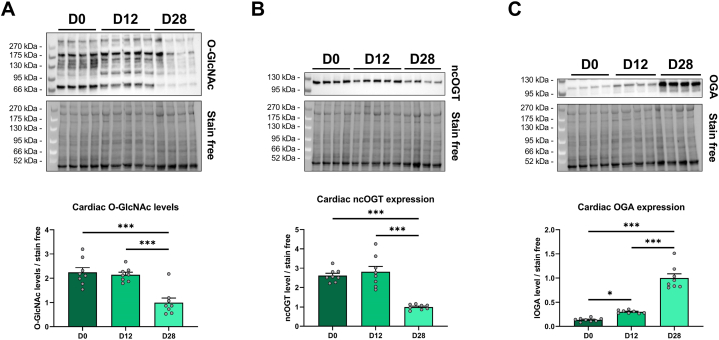
O-GlcNAcylation levels and O-GlcNAc associated enzymes throughout postnatal development. Evaluation by Western blot of the O-GlcNAcylation levels (**A**), ncOGT (**B**) and OGA (**C**) expression in the heart after the birth (D0), before (D12) and after weaning (D28). Quantification was performed in relation to stain free. Results are expressed as mean ± SEM. n = 8. Statistical significance was assessed by one way ANOVA with Uncorrected Fisher's LSD test. *: p < 0.05; ***: p < 0.001. Full blots are shown in Supplemental Figs. S1–S3.

Cardiac O-GlcNAc levels are decreased by a 2-fold at 28 days compared to both D0 and D12 group (D0: 2.2 ± 0.2; D12: 2.1 ± 0.1; D28: 1.0 ± 0.2; p < 0.05) (Fig. 3A). This modification can be explained by the fall of the expression of ncOGT at 28 days (D0: 2.6 ± 0.1; D12: 2.8 ± 0.3; D28: 1.0 ± 0.1; p < 0.05) and the raise of OGA by a 7-fold in the D28 group compared to D0 (D0: 0.13 ± 0.01; D12: 0.30 ± 0.01; D28: 1.00 ± 0.09; p < 0.05) (Fig. 3B and C).

### 3.2 Dietary modification impacts ncOGT and OGA without affecting O-GlcNAc levels

Results obtained and depicted on Fig. 3 suggest that O-GlcNAc levels and the expression of associated enzymes are linked to the suckling to weaning transition occurring between 12 and 28 days of age. The suckling to weaning transition was abolished by feeding rats with delayed weaning diet to unravel its potential impact on O-GlcNAc levels and the expression of associated enzymes.

**Fig. 4:**
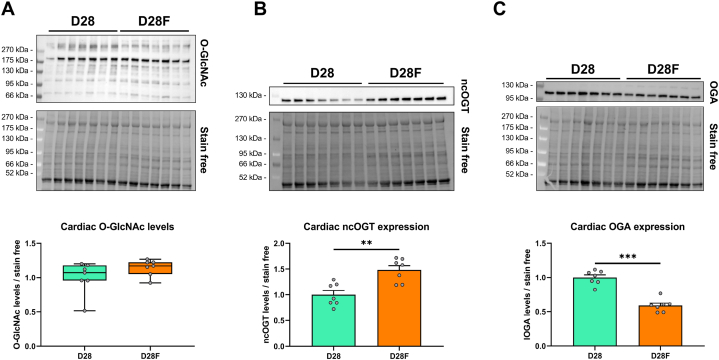
Impact of delayed weaning diet on O-GlcNAcylation and associated enzymes Evaluation by Western blot of the O-GlcNAcylation levels (**A**), ncOGT (**B**) and OGA (**C**) expression in the heart between standard alimentation (D28) and delayed weaning diet (D28F). Quantification was performed in relation to stain free. Results are expressed as mean ± SEM. n = 7. Statistical significance was assessed by a Mann-Whitney test (O-GlcNAc levels) or *t*-test (ncOGT and OGA). **: p < 0.01; ***: p < 0.001. Full blots are shown in Supplemental Figs. S4–S6.

Interestingly, the diet modification does not impact cardiac O-GlcNAc levels (D28: 1.00 ± 0.09; D28F: 1.14 ± 0.05) (Fig. 4A). The expression of ncOGT and OGA is respectively increased and decreased in the D28F group (ncOGT: D28: 1.00 ± 0.08; D28F: 1.48 ± 0.09; OGA: D28: 1.00 ± 0.04; D28F: 0.59 ± 0.03; p < 0.05) (Fig. 4B and C). These results suggest that adaptative mechanisms leading to ncOGT and OGA expression modifications are implemented to maintain O-GlcNAc levels.

### 3.3 UAP1 and UAP1L1 cannot explain changes in O-GlcNAcylation levels throughout postnatal development

**Fig. 5:**
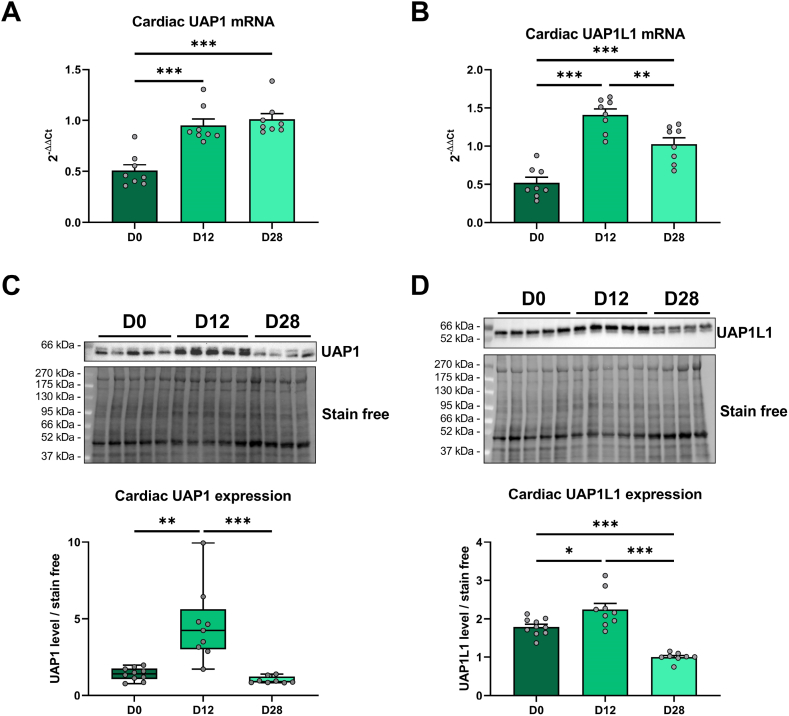
UAP1 and UAP1L1 mRNA and protein expression throughout postnatal development Evaluation by qPCR and Western blot of UAP1 mRNA (**A**) and protein (**C**) expression and UAP1L1 mRNA (**B**) and protein (**D**) expression in the heart after the birth (D0), before (D12) and after weaning (D28). Western blot quantification was performed in relation to stain free. Results are expressed as mean ± SEM. n = 8–10. Statistical significance was assessed by one way ANOVA with Uncorrected Fisher's LSD test (**A**, **B** and **D**) or a Kruskal-Wallis test with Uncorrected Dunn's test (**C**). *: p < 0.05; **: p < 0.01; ***: p < 0.001. Full blots are shown in Supplemental Figs. S7–S8.

UAP1 is the last enzyme of the HBP for the synthesis of the UDP-GlcNAc. UAP1 like 1 (UAP1L1) share 60 % of homology with UAP1 but lacks its enzymatic activity. However, UAP1L1 interacts with OGT and can modulate its activity [26]. We wanted to assess the variation of mRNA and protein expression of both UAP1 and UAP1L1 in the heart. Cardiac UAP1 and UAP1L1 mRNA are increased at D12 and D28 compared to D0 (UAP1: D0: 0.50 ± 0.06; D12: 0.95 ± 0.06; D28: 1.01 ± 0.06; UAP1L1: D0: 0.52 ± 0.07; D12: 1.41 ± 0.08; D28: 1.02 ± 0.08; 2^−ΔΔCt^). UAP1L1 mRNA is decreased at D28 compared to D12 in the heart (Fig. 5A and B). At the protein level, UAP1 is increased at D12 compared to D0 and then decreased at D28 (D0: 1.4 ± 0.1; D12: 4.6 ± 0.8; D28: 1.0 ± 0.1). Cardiac UAP1L1 protein expression showed a similar profile to the mRNA (D0: 1.8 ± 0.1; D12: 2.2 ± 0.2; D28: 1.0 ± 0.1) (Fig. 5C and D).

### 3.4 Diet modification does not impact UAP1 and UAP1L1 protein expression

**Fig. 6:**
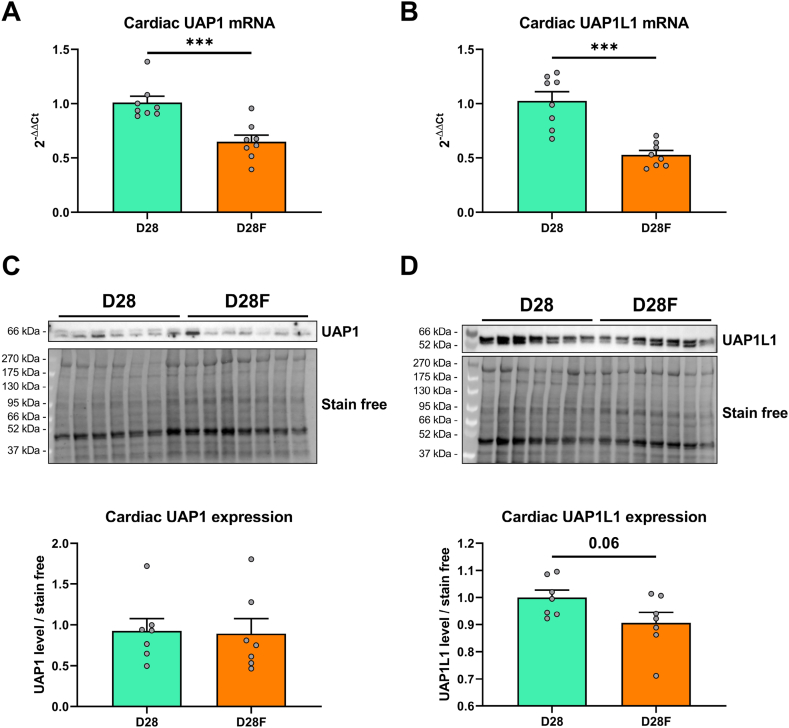
**Impact of delayed weaning diet on UAP1 and UAP1L1 mRNA and protein expression** Evaluation by qPCR and Western blot of UAP1 mRNA (**A**) and protein (**C**) expression and UAP1L1 mRNA (**B**) and protein (**D**) expression in the heart between standard alimentation (D28) and delayed weaning diet (D28F). Statistical significance was assessed by unpaired *t*-test. Western blot quantification was performed in relation to stain free. Results are expressed as mean ± SEM. n = 6–8; ***: p < 0.001. Full blots are shown in Supplemental Fig. S9.

O-GlcNAcylation levels are unchanged with dietary intake change, conspicuously due to a modulation of the expression of enzymes involved in the turn-over of this modification (Fig. 4). To decipher the potential role of both UAP1 and UAP1L1 in the adaptive response to diet modification, mRNA and protein expression have been evaluated. UAP1 and UAP1L1 mRNA expression are decreased in the D28F group (UAP1: D28: 1.01 ± 0.06; D28F: 0.65 ± 0.06; UAP1L1: D28: 1.02 ± 0.08; D28F: 0.52 ± 0.04; 2^−ΔΔCt^) (Fig. 6A and B). Interestingly, cardiac protein expression remains unchanged between the two groups (UAP1: D28: 1.0 ± 0.2; D28F: 0.8 ± 0.2; UAP1L1: D28: 1.0 ± 0.1; D28F: 0.9 ± 0.1) (Fig. 6C and D). These data are in accordance with previous results (Fig. 5) and suggest that neither UAP1, nor UAP1L1 are the main regulator of the O-GlcNAcylation levels.

### 3.5 Transcriptomic landscape evolves throughout postnatal development and changes with the delayed weaning diet

#### 3.5.1 Two by two comparisons reveal modification of gene expression and related biological processes

Our results demonstrate that O-GlcNAc levels are strictly maintained even if substrate metabolism is modified with a delayed weaning diet. We wanted to explore if the regulation goes through transcriptome modifications to maintain precise O-GlcNAc levels throughout the postnatal development period. Comparisons of time point were made to compare a normal condition of development (D0 vs D12 and D12 vs D28) or the abolishment of the weaning-dependent metabolic transition (D28 vs D28F).

**Fig. 7:**
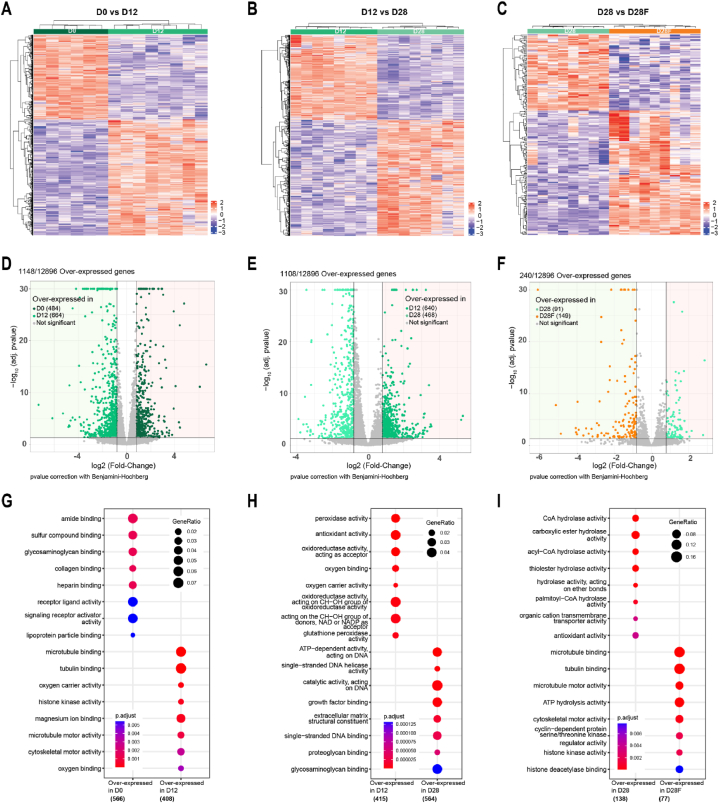
Impact of development and delayed weaning diet on heart gene expression Transcriptome analyses were made from whole heart from rats collected after the birth (D0), before (D12) and after weaning (D28), after weaning with delayed weaning diet (D28F). Differentially expressed genes were selected if (log2Ratio() > 0.8) or (log2Ratio() < -0.8) and p-value<0.05(BH test and correction). Each comparisons analysis is visualized thanks to Heatmaps (**A**, **B**, and **C**, respectively), and volcano-plots (**D**, **E**, and **F**, respectively). Top represented GO-BP are presented with dot-blot(**G**,**H**, and **I**, respectively).

3′ SRP approach allows the detection of 12896 cardiac genes (Supplemental Table 1). Among them, differentially expressed genes (DEGs) were selected by meeting our quality criteria, based on Fold-Change (FC) and the p-adjusted value as follows: (Log2 (FC) < −0.8 or > 0.8 and p-adjusted < 0.05). Overall, DEGs visualization on heatmap showed clustered samples within groups, this clustering and group separation have also been observed and confirmed on a Principal component analysis (Supplemental Fig. 10). As a result, 1148 DEGs have been found differentially expressed between D0 (42 % overexpressed) and D12 (58 % overexpressed) (Fig. 7A–D). For D12 *vs* D28, 1106 DEGs passed the selected criteria, with 58 % and 42 % overexpressed at D12 and D28, respectively (Fig. 7B–E). Finally, 240 DEGs have been identified after the comparison of D28 and D28F, 38 % were overexpressed in the D28 group and 62 % were overexpressed in the D28F group (Fig. 7C–F). Interestingly, biological processes associated with the first comparison (D0-D12) show that 12 days after birth, transcriptome from rat's heart express less genes involved in receptors activity and binding of glycans and overexpress genes related to cytoskeletal organization and oxygen transfer or use (Fig. 7G). The latter process is once again found to be overexpressed with more processes oriented to the mitochondrial management of oxidative metabolism and metabolites at D12 when compared to D28 (Fig. 7H). Finally, it has been found at D28 that the regulation of DNA structure and DNA binding to other components were processes highly enriched. For the last comparison (D28-D28F), heart from rats fed with the delayed weaning diet show less enriched processes implicated in energy metabolism and Acetyl-CoA metabolic processes than rats fed with normal diet at D28 and present overexpressed genes implicated in DNA and oxidative regulations. Interestingly, GFAT1, GFAT2, OGT and OGA were not differentially expressed in our groups, and no gene ontology related to O-GlcNAcylation or HBP were found (Fig. 7I).

#### 3.5.2 DEGs expression profile analysis highlights distinctive gene cluster expression signatures

**Fig. 8:**
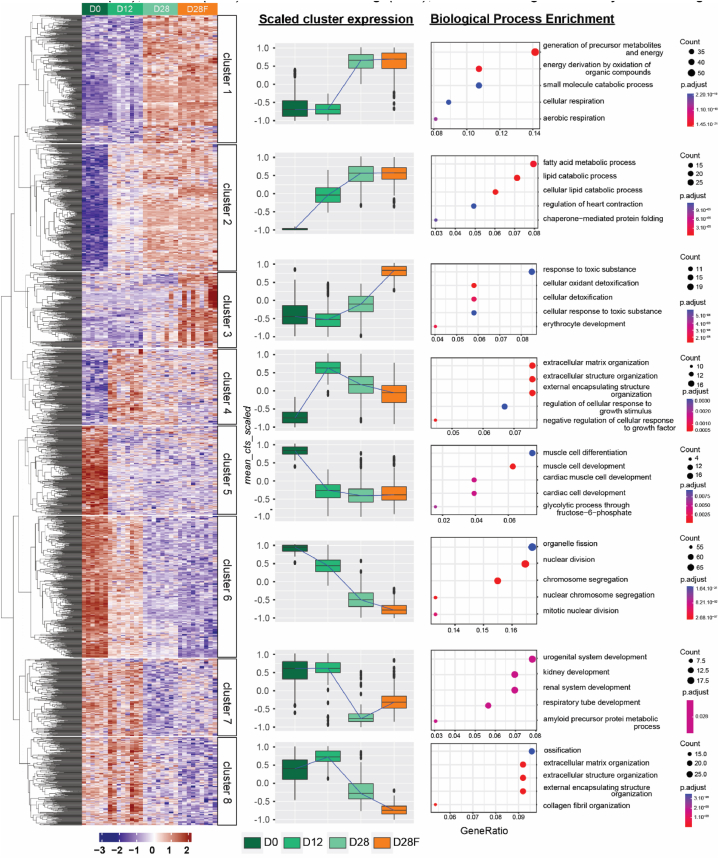
Gene expression profile throughout postnatal development and with delayed weaning diet All genes selected by groups comparisons were collided and their scaled expressions were plot in a heatmap after clustering (first column). Mean count scaled of genes per cluster in function of groups were calculated and plotted to visualize the trajectory of expression of these cluster in heart from rats after the birth (D0), before (D12) and after weaning (D28), after weaning with delayed weaning diet (D28F) (Second column). Most representative functions associated with each cluster are highlighted by a Gene Ontology (Biological Process) analysis (Third column).

The previous approach of 2 by 2 comparisons does not allow to have a proper idea of the expression profile overtime and what processes are tightly regulated during development. In order to visualize how genetic expressions are evolving with our conditions, DEGs shared by all the groups were pooled together. From this gene set, hierarchical clustering of gene has been performed and individuals were classified by groups as represented on the expression heatmap (Fig. 8) (Supplemental Table 2). From the eight identified clusters, mean expression of DEGs per groups have been evaluated to decipher the expression profile differences between groups and depicted in Fig. 8. Over-representation analysis was performed for each individual cluster to link their gene expression profiles with the most represented biological functions or processes. This approach shows that indeed each cluster presents a specific gene expression profile which is associated to different biological processes or pathways (GO, KEGG, Reactome; Supplemental Table 3).

Based on this approach, two gene expression signatures have been identified throughout postnatal development. Genes related to metabolism (cluster 1) and more particularly lipid metabolism (cluster 2) as well as genes involved in cellular response to toxic or stress (cluster 3) and in extracellular organization (cluster 4) are increased with the development. Conversely, DEGs implicated in cellular development (cluster 6) and tissue specialization (cluster 5–8) are less expressed with postnatal development (Fig. 8).

Surprisingly, the replacement of the suckling to weaning transition shows no impact (clusters 1, 2, 5) or a slight impact on gene expression profiles at D28 (clusters 4, 6, 7). Delayed weaning diet however seems to trigger by itself, without the influence of development, an increase in transcript level of genes implicated in the cellular detoxification processes, handle of oxidative stress and extracellular organization (clusters 3 and 8) (Fig. 8).

The last step of our approach was to focus more on O-GlcNAcylation related genes. To achieve this, genes involved in HBP and O-GlcNAcylation moiety management were screened among all genes found as differentially expressed over the postnatal development stages and during the metabolic transition. Interestingly only UAP1L1 (UDP-N-Acetylglucosamine Pyrophosphorylase 1 Like 1) is significantly more expressed over the postnatal period in our condition (overexpressed at D12 *vs* D0). Surprisingly and despite significant modifications in OGT and OGA protein expression throughout development and with delayed weaning diet, no significant changes in genetic expression were highlighted, suggesting a post-transcriptional regulation of these enzymes.

## 4 Discussion

In this study, we demonstrated for the first time that changes in cardiac O-GlcNAc levels throughout postnatal development are not associated with changes in OGT and OGA transcripts. Moreover, we showed that O-GlcNAcylation levels are strictly regulated throughout postnatal development, suggesting a role in heart development and maturation.

Substrate availability has been described as impacting O-GlcNAcylation levels. Our work showed that changes in diet composition are not impacting O-GlcNAcylation levels. High-fat diet was associated in previous studies with an increase in O-GlcNAc levels in several tissue like cerebral arteries, liver, retina, aorta, white adipose tissue, intestine, skeletal muscle and heart at an older age [[27], [28], [29], [30], [31], [32], [33], [34], [35], [36]]. Interestingly, all these high fat diet studies in animals were carried out after the suckling to weaning transition which is in fact a key period for cardiac development. Protein level of ncOGT and OGA are respectively increased and decreased under high fat diet potentially to maintain physiological levels of O-GlcNAc. That suggests unlike in adults that O-GlcNAcylation is not affected by the high-fat diet during development. The fine regulation of O-GlcNAcylation throughout cardiac postnatal development suggests its implication in heart developmental processes.

Only few studies focused on the role of the O-GlcNAcylation in heart development despite OGT and OGA are essential for organisms viability and widely described as involved in adult heart pathophysiology [[37], [38], [39]] [[37], [38], [39]] [[37], [38], [39]]. Embryonic cardiospecific deletion of OGT has been shown to induce dilated cardiomyopathy and multiple cardiac defects, associated with changes of the transcriptome [40]. According to these previous results, Xiong *et al.* showed that deletion of OGT in both cardiomyocytes and smooth muscle cells was associated with dilated cardiomyopathy, pathological remodeling of the heart and reduction of contractile genes expression in vascular smooth muscle cells [41]. Interestingly, loss of OGA catalytic activity has also been reported as leading to cardiac defects (e.g., interventricular septum, 3rd and 4th ventricle, defect in tricuspid and mitral valves) in E18.5 mouse embryo [42]. As well as being involved in development, O-GlcNAcylation is thought to play a major role in heart maturation. Watson *et al.* evaluated the impact of KO of OGT specifically in cardiomyocytes. They highlighted that just after weaning, hearts from mice KO OGT presented a cardiomyopathy (increased ventricular volumes, decreased ejection fraction and cardiac output) as well as ventricular remodeling (increased fibrotic area, apoptosis and cardiomyocytes size). They also point out that OGT deletion leads to changes in transcription of metabolic genes [43]. Finally, excessive O-GlcNAcylation could also impact heart function and development. Interestingly the overexpression of cardiac OGT in transgenic mice also showed to have adverse effects leading to sever dilated cardiomyopathy by increasing O-GlcNAc levels, effects that were counteracted with an overexpression of OGA [44]. Kim *et al.* demonstrated that expression of *Nkx2.5*, an essential transcription factor for cardiac development, dropped under high O-GlcNAcylation levels and thus suppressed the spontaneous cardiogenesis in embryonic stem cells [45]. Combined with our results that pinpoint the necessity to have a certain cardiac level of O-GlcNAcylation at a given developmental stage, even during the post-natal stage of cardiac development, these data are strong evidence of the pivotal role play by O-GlcNAcylation in heart development and maturation and notably its impact on gene expression.

Understanding the regulation of O-GlcNAcylation, more specifically through the identification of the main regulatory element, is one of the current challenges. We demonstrated that the O-GlcNAc levels decrease throughout the first month after birth according to decrease in ncOGT and increase in OGA. Few studies have demonstrated the existence of feedback of O-GlcNAcylation levels on the expression of the OGT/OGA enzyme couple. In 2014, Zhang *et al.* demonstrated that OGA expression was sensitive to change in O-GlcNAc homeostasis with an increase in OGA mRNA when O-GlcNAc levels were stimulated [46]. Park *et al.* highlighted a decrease in OGT expression in response to O-GlcNAc elevation through an intronic splicing silencer sequence [47]. In contrast to these two studies where O-GlcNAcylation levels are artificially modulated, our results demonstrate that in physiological conditions, these regulatory mechanisms are not applied. Our transcriptomic approach revealed no changes of *OGT* or *MGEA5* (encoding OGA) transcript level. These data strongly suggest either or both the existence of a post-transcriptional regulation or splicing that cannot be detected with 3′SRP transcriptomic and the existence of a sensor which regulates O-GlcNAc levels through mechanisms depending on the conditions (e.g. physiological *vs* pathological variations). We demonstrated in a previous study that none of the hexosamine biosynthesis pathway metabolites quantified by mass spectrometry (glucose-6-phosphate, fructose-6-phosphate, fructose-1,6-bisphosphate, glutamine and UDP-GlcNAc) reflected the variation in global O-GlcNAcylation levels observed in the heart. The latter result is suggesting that none of these metabolites were the main regulatory element of O-GlcNAcylation [19]. The UDP-GlcNAc is synthetized by UAP1. UAP1L1 presenting a 60 % sequence homology to UAP1, has no enzymatic activity but has the ability to potentiate OGT activity by interacting with it [48]. In human hepatoma cells, Lai *et al.* highlighted that knockdown of UAP1 and UAP1L1 markedly reduced O-GlcNAcylation levels. The authors also suggested that UAP1 and UAP1L1 impact O-GlcNAcylation levels via distinct mechanisms [26]. Interestingly, *UAP1L1* is the only O-GlcNAc related gene differentially expressed in our conditions. We demonstrated for the first time that both UAP1 and UAP1L1 protein expression varies throughout postnatal development with an increase at D12 before decreasing at D28 which is consistent with the decrease in O-GlcNAcylation levels at D28. Interestingly, while UAP1 and UAP1L1 mRNA are decreased with delayed weaning diet, their protein expression remains unchanged.

## 5 Conclusion

In conclusion, this study demonstrated that changes in gene expression associated with delayed weaning diet are not explained by O-GlcNAcylation as O-GlcNAc levels are unmodified. Overall, these results pinpointed that O-GlcNAcylation is finely regulated. Its regulation depends on several metabolites (UDP-GlcNAc, glutamine) and enzymes (OGT, OGA, UAP1, UAP1L1) that can interact together, making O-GlcNAcylation understanding even more difficult. The comprehension of how O-GlcNAcylation is regulated remains a major perspective to elucidate.

## Ethics statement

The study was conducted in respect with the guidelines and recommendation of the ethics committee in charge of animal experimentation of the Pays de la Loire, the French law on animal welfare, the EU Directive 2010/63/EU for animal experiments, and the National Institutes of Health (NIH) Guide for the Care and Use of Laboratory Animals (NIH Pub. No. 85-23, revised 2011).

## Funding

This work was supported by “10.13039/501100014262Société Française d’Anesthésie et de Réanimation” (Paris, France), “Fondation d'entreprises Genavie” (Nantes, France), “10.13039/501100003100Fédération française de cardiologie” (France), “Agence nationale de la recherche” (20-ASTC-0032-01-hErOiSmE) (Paris, France) and “10.13039/501100006021Direction Générale de l’Armement” (Paris, France). Thomas Dupas was supported by grants from 10.13039/501100006021Direction Générale de l’Armement (DGA) (France) and Région des Pays de la Loire during his PhD. Antoine Persello was supported by grants from InFlectis BioScience, France during his PhD.

## Data availability

All the data are included in the article and supplementary material.

## CRediT authorship contribution statement

**Antoine Persello:** Writing – review & editing, Writing – original draft, Investigation, Conceptualization. **Thomas Dupas:** Writing – review & editing, Writing – original draft, Investigation, Conceptualization. **Amandine Vergnaud:** Writing – review & editing, Investigation. **Angélique Blangy-Letheule:** Writing – review & editing, Investigation. **Virginie Aillerie:** Writing – review & editing, Investigation. **Angélique Erraud:** Writing – review & editing, Investigation. **Yannick Guilloux:** Writing – review & editing, Investigation. **Manon Denis:** Writing – review & editing, Investigation. **Benjamin Lauzier:** Writing – review & editing, Funding acquisition, Conceptualization.

## Declaration of competing interest

The authors declare that they have no known competing financial interests or personal relationships that could have appeared to influence the work reported in this paper.
